# European Medicines Agency Policy 0070: an exploratory review of data utility in clinical study reports for academic research

**DOI:** 10.1186/s12874-019-0836-3

**Published:** 2019-11-05

**Authors:** Jean-Marc Ferran, Sarah J. Nevitt

**Affiliations:** 1 Qualiance ApS, Copenhagen, Denmark; 2 Department of Biostatistics, University of Liverpool, Block F, Waterhouse Building, 1-5 Brownlow Street, Liverpool, L69 3GL UK

**Keywords:** Policy 0070, Regulatory, Anonymised, Clinical study reports, Data utility, Review

## Abstract

**Background:**

Clinical study reports (CSRs) have been increasingly utilised within academic research in recent years. European Medicines Agency (EMA) Policy 0070 ‘Phase 1,’ which came into effect in January 2015, requires the publication of regulatory documents such as CSRs from central applications in an anonymised format. EMA Policy 0070 requires sponsors to demonstrate careful consideration of data utility within anonymised CSRs published within the scope of the policy, yet the concept of data utility is not clearly defined in the associated anonymisation guidance.

**Objective:**

To review the use of data from CSRs in published academic research and to hypothesise the potential data utility of CSRs anonymised under the objectives of EMA Policy 0070 for future academic research.

**Methods:**

Review of the objectives, research methodologies and findings of academic research reports using unpublished data from CSRs (prior to EMA Policy 0070). Semi-structured interviews with authors of academic research reports, including questions related to data utility of anonymised CSRs published under EMA Policy 0070.

**Results:**

Thirteen academic research reports were identified and reviewed. The research purposes ranged from assessment of reporting bias, comparison of methods and results with published data sources, detailed evaluation of harms and adverse events, re-analysis and novel analyses including systematic reviews and meta-analysis. All of the examples identified required access to the methods and results sections of CSRs (including aggregated summary tables) and research purposes relating to evaluation of adverse events also required access to participant narratives. Retaining anonymised participant narratives relating to interventions, findings and events, while maintaining an acceptably low risk of participant re-identification, may provide an important gain in data utility and further understanding of drug safety profiles.

**Conclusions:**

This work provides an initial insight into the previous use of CSR data and current practices for including regulatory data in academic research. This work also provides early guidance to qualitatively assess and document data utility within anonymised CSRs published under EMA Policy 0070.

## Background

Clinical Study Reports (CSRs) represent a wealth of information related to design, conduct and analysis of clinical trials, in addition to more comprehensive trial results compared to publicly available sources such as journal manuscripts and clinical trial registries. Doshi et al. 2013 [1] refer to CSRs as a “hitherto mostly hidden and untapped source of detailed and exhaustive data on each trial” and make reference to the concept of a “compression factor,” defined as the ratio of CSR page length compared to the page length of the journal publication of the same trial, ranging from 1 to 8805 based on the review of 78 CSRs [1].

Previous work has highlighted the impact of selective outcome reporting [2, 3], in that the data and results published within a journal manuscript may be incomplete or misleading, and the biases that originate from this selective reporting. Increasingly, researchers undertaking novel secondary analyses of clinical trial data such as systematic reviews and meta-analyses are seeking access to previously confidential regulatory documents, such as CSRs. These documents are used as a means of appraising, revaluating and reducing the impact of any misreporting or selective reporting bias, generating more complete and reliable information and investigating clinical questions which could not have previously been considered using published data sources alone [4–16].

The European Medicines Agency (EMA) policy on the publication of clinical data for medicinal products for human use (EMA Policy 0070 ‘Phase 1’, herein referred to as EMA Policy 0070), allowing global access to regulatory documents for non-commercial purposes, came into effect in January 2015. The policy requires ‘clinical reports’ (defined as clinical overviews, clinical summaries, and CSRs together with the following appendices to the CSRs: protocol and protocol amendments, sample case report form and documentation of statistical methods (clinical trial statistical analysis plan (SAP)) from central regulatory applications to be published in an anonymised portable document format, such that the risk of re-identification of trial participants from the information available contained within these documents, including any retained narrative information relating to individual participants, is deemed to be acceptably low for public disclosure of data [17].

The concept of ‘Data Utility’ appears to be an important criterion for anonymised CSRs to meet the objectives of EMA Policy 0070. Reference is made within several sections of EMA Policy 0070 anonymisation external guidance and within the ‘Anonymisation Report’ template that the applicant must demonstrate careful consideration of “the impact of the anonymisation methodology used on data utility [17].” However, despite the apparent importance of this criterion, the EMA Policy 0070 external guidance [17] does not define or quantify the utility of anonymised CSRs (and “Data Utility” is absent within section “3. Definitions”) [17].

The Organization for Economic Co-operation and Development (OECD) propose the following definition for ‘Data Utility’: “*A summary term describing the value of a given data release as an analytical resource. This comprises the data’s analytical completeness and its analytical validity. Disclosure control methods usually have an adverse effect on data utility. Ideally, the goal of any disclosure control regime should be to maximise data utility whilst minimising disclosure risk. In practice disclosure control decisions are a trade-off between utility and disclosure risk* [18].”

Clearly, the ‘analytical completeness and analytic validity’ of anonymised CSR data, and therefore the relative ‘data utility’ of anonymised CSRs made public under EMA Policy 0070 are directly dependent on the consumers of the data and their reasons or purposes for using this data.

Therefore the objective of this work is to review the previous use of data from CSRs in academic research and with these research purposes in mind, to hypothesise the potential data utility of CSRs anonymised under the objectives of EMA Policy 0070.

## Methods

This paper begins with a short reflection of requests made under EMA Policy 0043, a policy which enables requests for access to regulatory documents (related to medicinal products for human and veterinary use), followed by a summary and discussion of previous research conducted using CSR data [4–16].

### Insights from EMA policy 0043

**Fig. 1:**
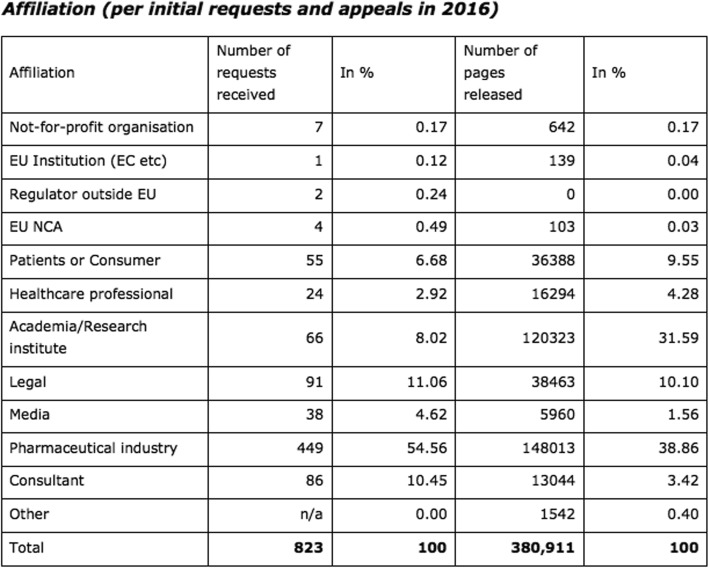
Policy 0043 documents requests per affiliation

Source: Annexes to the annual report of the European Medicines Agency 2016, Annex 19 [19].

An EMA Policy on access to documents (related to medicinal products for human and veterinary use), known as ‘EMA Policy 0043’, came into effect in 2010. Requesting documents through EMA Policy 0043 is a controlled process and requesters must identify themselves and their affiliation but it are not required to document the purpose of their request as part of the requesting process. Figure 1 summarises the number of requests and number of pages released per affiliation and was made public in 2016 [19].

1. ‘Legal’ and ‘Consultant’ requesters refers to mostly professionals from or contracted by the pharmaceutical industry. The category “Pharmaceutical industry” both includes companies from the innovative and generic industry; no distinction was made.
2. The request process of EMA Policy 0043 includes an appeal process in the case of an initial rejection

It should be noted that the scope of EMA Policy 0043 is not limited to requests for clinical reports (i.e. CSRs and other documents related to clinical trial conduct and results (such as clinical trial protocols and SAPs) and a range of other documents such as commercial, technical or legal documents as well as meeting minutes could also be requested. A breakdown of the information within Fig. 1 relating to the type of documents requested was not available.

Grouping together the first seven rows to represent ‘Non-commercial’ requests from “Not-for-profit organisation” to “Academia/Research institute”, the number of requests received represent 18.6% of total requests and ‘Commercial’ requests from ‘Legal’, ‘Pharmaceutical Industry’ and ‘Consultant’ represents 76% of total requests. However, when comparing the number of pages released, the ‘Non-Commercial’ and ‘Commercial’ groups are approximately equivalent with 52.3 and 45.6% respectively. This shift is mainly explained by requests for documents with a large number of pages (likely to be clinical reports such as CSRs, trial protocols, trial SAPs) by Academia/Research institutes (31.59%) while other subgroups may be more likely to request other documents (such as technical documents or meeting minutes) that represent fewer pages. It is also of note that requests from “Patients or Consumers” represent 6.68% of all requests and 9.55% of pages released.

We note that direct extrapolation of these figures from EMA Policy 0043 (a controlled process covering all types of documents) to EMA Policy 0070 (public access to clinical reports as part of a central application) is not recommended and the type of documents requested by each consumer group via EMA Policy 0043 is merely an assumption. Nonetheless, the affiliations described within Fig. 1 provide an indication of who the main data consumers of EMA Policy 0070 may be and further research on EMA Policy 0043 requests, particularly relating to the types of documents requested by each data consumer group, could help in understanding the likely consumers of EMA Policy 0070 and their purposes.

## Results

### Use of clinical study reports in academic research

A number of examples of journal publications using CSRs in academic research [4, 8–11, 14, 15] were cited within a conference presentation delivered by Tom Jefferson in 2017 [20] and within a related published report in 2018 [21]. This section summarises and discusses the research purposes and methodology applied within these examples, in addition to a recently published Cochrane Review involving SJN which has made use of CSR data in analysis [16].

Some of the research objectives from these examples, outlined in general terms including the sections of the CSRs that may be required for the research, are summarised in Table 1. We further discuss some of the research objectives outlined within the examples within this section. Further specific details of the all regulatory data sources provided for the research, the methods employed within each example and the main findings of the research are summarised in Additional file 1: Table S1.

**Table 1:** Examples of the objectives of research using CSR data

Objective	CSR section(s) that may be required	Examples
Assessment of reporting and evaluation of bias	Methods, results (aggregate summary tables and text), narratives, participant listings	Eyding et al [4], Schroll et al [9], Hodkinson et al [10], Jefferson et al [11], Vedula et al [15]
Comparison of methods and/or results (including adverse events) with data registries or manuscripts	Methods, results (aggregate summary tables and text)	Eyding et al [4], Le Noury et al [8], Schroll et al [9], Hodkinson et al [10], Jefferson et al [11], Maund et al [12–14], Vedula et al [15], Nevitt et al [16]
Detailed evaluation of harms and adverse events	Results (aggregate summary tables and text), narratives, participant listings	Eyding et al [4], Maund et al [12–14]
Systematic review and meta-analysis (evidence synthesis)	Methods, results (aggregate summary tables and text), narratives	Eyding et al [4], Jefferson et al [11], Maund et al [14], Nevitt et al [16]
Re-analysis (*repeating original analysis*)	Methods, results (aggregate summary tables and text), narratives, participant listings	Le Noury et al [8] (also using individual participant data)^a^
Re-analysis (*different method or objective to the original analysis*)	Methods, results (aggregate summary tables and text), narratives, participant listings	Maund et al [14], Nevitt et al [16]

^a^In this example, Le Noury et al [8], re-analysis was conducted using both individual participant data (IPD), requested via data sharing platform clinicalstudydatarequest.com [22] in addition to supporting information from CSRs and case report forms

We note that many of the research purposes outlined in Table 1 overlap and are not intended to be an exhaustive list; rather a general summary of a sample of published research using CSR data at the time of writing. It must be noted that the examples discussed within this section are a ‘selective sample’ of academic work which has mostly shown changes in conclusions, particularly regarding harms of drugs, when analysing or re-analysing clinical trial data using CSRs and the methodological approaches taken by some of the academic research groups have been challenged by the pharmaceutical companies in question (see Additional file 1: Table S1). This sample should not be considered completely reflective of academic objectives for accessing regulatory documents or a comprehensive list of all research using CSRs (which is likely much wider as indicated by the number of requests from Fig. 1) The selective nature of the sample summarised here must be taken into account when interpreting the findings within the context of all published research making use of unpublished regulatory documents.

### Assessment of reporting and evaluation of bias

A common reason for using unpublished information, such as the trial protocols and CSRs of specific trials to access to detailed trial methodology and comprehensive results, is to perform an appraisal, assessment and evaluation of any bias in the trial design, misreporting or any selective outcome reporting bias in journal manuscripts [2, 3]. It is recommended within the *Cochrane Handbook for Systematic Review of Interventions* that selective outcome reporting should be appraised within Cochrane Reviews by comparing trial protocols with published reports [23] and examples identified within this review have also shown the use of CSRs for identifying misreporting or selective outcome reporting.

For example, by comparing the information in CSRs and other regulatory documents (such as protocols) to journal publications for 20 trials of Gabapentin, Vedula et al [15] identified selective outcome reporting for trials of off-label use of gabapentin which ‘threatens the validity of evidence for the effectiveness of off-label interventions’. Eyding et al [4] also discovered that published data overestimated the benefit of reboxetine versus placebo by up to 115% and reboxetine versus SSRIs by up to 23%, and also underestimated harm compared to the information presented in CSRs and other regulatory documents. Similarly, by comparing publications of Orlistat trials to their corresponding CSRs, both Schroll et al [9] and Hodkinson et al [10] identified that journal publications provided ‘insufficient’ information on harms outcomes of clinical trials and in some cases the authors noted ‘important disparities’ in the definitions and numbers of adverse events were reported across different documents relating to the same trial.

### Detailed evaluations of harms and adverse events

It is mandatory that a summary of adverse events (AEs) occurring during a trial is made public via registries such as ClinicalTrials.gov or EudraCT [24]. However, a number of examples have made use of the more detailed harms and AE information available within CSRs, both in the context of appraising ‘selective outcome reporting’ as outlined in the section above [4, 9–11, 15] as well as for novel analyses of previously unpublished harms and AE information [11–14].

For example, Maund et al [12, 13] present methodological investigations relating to the conclusions that can be drawn from reading summary tables of AEs which are usually dictionary coded, compared to reading verbatim descriptions in the narratives. Their illustrative example of nine trials of duloxetine for the treatment of major depressive order shows that coded events and narratives within CSRs suggest different numbers of events related to suicide and the authors conclude that in this case the narratives are more informative and coded events in summary tables may be misleading and may not fully capture the true nature of the event. Specifically:

“…*narratives of adverse events can provide additional information, including original investigator reported adverse event terms, which can enable a more accurate estimate of harms* [13].”

*“Using the patient’s trial identification number we were able to reconcile data reported in the patient listings with those in the narrative. Secondly, using data (treatment assignment, coded term, and timing of event) from the patient listings and narratives, we were able to reconcile data from these two formats with the data in summary tables* [13]*.”*

A related publication from Maund et al [14], presents a meta-analysis relating to the benefit and harms of duloxetine for the treatment of stress urinary incontinence, with a specific interest in harms related to suicide and violence, in response to FDA concerns about an association between duloxetine and these serious adverse events. The authors use data from CSRs (summary tables and narratives) to perform meta-analysis of four trials and compare their analysis to the results of a Cochrane review of the same topic conducted only with data available in the public domain (i.e. from trial publications). The two pooled analyses come to the overall same conclusion that clinical benefits of duloxetine for stress urinary incontinence do not outweigh the potential harms, but the two analyses consider different outcomes sets and show slightly different results for common outcomes. Notably, the authors note that the analysis of CSR data has allowed more detailed considerations of specific AEs which would not have been possible without access to CSRs and narratives.

For example, *“one patient had a “nervous breakdown,” which was coded as mental disorder, and another patient reported “feeling drugged,” which was coded as somnolence. In addition, 5 patients, all receiving duloxetine, experienced a total of 8 events that were mentioned only in the narrative text* [14]*.”*

These publications highlight the potential utility of the participant narratives and participant listings for novel analyses relating to AEs and further understanding of drug safety profiles beyond summary statistics of dictionary-coded events. It must be noted that participant listings are out-of-scope for ‘Phase 1’ of EMA Policy 0070 and work conducted in November 2017 showed that the majority of CSRs published under EMA Policy 0070 have ‘fully redacted’ participant narratives [25], likely due to concerns regarding the risk of re-identification from narrative information relating to a specific participant and technical challenges to retrospectively anonymise unstructured data (i.e. narratives and body text), despite EMA Policy 0070 external guidance specifying that case narratives should be anonymised rather than removed [17]. With the valid re-identification concerns in mind, primarily, the ability to follow the ‘journey’ of a participant through a trial, particularly preserving sequences and distances between interventions, findings and events (and access to different definitions of events, including investigator reported terms) has clear advantages for the ‘utility’ of CSR data and is essential for detailed evaluations of harms outcomes, such as in the examples of Maund et al [12–14]. Secondarily, availability of demographics and medical history among others could also support more detailed analyses, including analyses relating to harms and AEs.

### Use of previously unpublished summary data for systematic reviews and meta-analyses

Previous work has shown that publicly available information (such as from journal publications and trial registries) of primary and secondary outcomes, as well as patient-relevant outcomes and harms, may not be sufficient [5–7, 11, 16], particularly for the objectives of a novel systematic review or meta-analysis [11, 16]. Additionally, the format that the summary results are provided in may not readily allow the inclusion of the information within meta-analysis; for example, where a measure of precision of the treatment effect is not published or if numerical results are not reported for all measurement times [16].

Therefore, in order to provide the most clinically informative and high quality systematic review and meta-analysis results as possible, researchers may wish to use unpublished summary (aggregated) data from one or more CSRs within systematic reviews and meta-analyses. In 2014, Jefferson et al [11, 27, 28] reported on the first Cochrane review to be based on all relevant full CSRs of a drug (oseltamivir), augmented by ‘regulatory comments’ (i.e. any other relevant information submitted to regulatory authorities for oseltamivir).

A CSR would typically contain more details about the choice of statistical method, interpretation of results and the full set of endpoints, results and statistics at all time points measured and therefore may provide a useful supplementary source of data for systematic reviews and meta-analysis. For example, a review of 101 CSRs conducted by Wieseler et al [5] shows that CSRs provided complete information on 78 to 100% of benefit outcomes (compared to 20 to 53% from publicly available sources), CSRs also provided considerably more information on harms and on patient-relevant outcomes such as outcomes describing morbidity, mortality, and health-related quality of life.

### Use of regulatory data in Cochrane reviews: current practice and attitudes

A survey was conducted among Cochrane authors between June and September 2016 regarding any experiences and opinions they had of using CSRs and ‘other regulatory documents’ (defined as any document produced by or held by a regulatory agency) in Cochrane Reviews [20, 21, 29].

There were 156 respondents out of 7181 invited Cochrane authors who had worked on a Cochrane review in the previous 2 years (around 2% response rate). Results of the survey showed only 10% of the 156 respondents used or requested regulatory data (mostly CSRs to supplement meta-analyses) from pharmaceutical companies directly or from regulatory agencies such as the EMA and the FDA. A further 5% had considered using regulatory data and the remaining 85% had not considered using regulatory data. Of those who had experience of using or requesting regulatory data, 80% believed that regulatory data should be used on Cochrane reviews, yet two thirds of these respondents who had accessed and included CSR data in Cochrane reviews mentioned barriers to using this data such as limited or restricted access to the data (such as not being able to print documents and download data) and lack of experience or skills required to interpret these detailed documents [29].

The proportion of individuals who believed that regulatory data should be used in Cochrane Reviews falls to 38 and 32% respectively for those who had considered or had not considered using regulatory data and results of the survey also showed that out of all respondents, only 12% claimed to know where to access regulatory information from clinical trials and 32% of respondents admitted to having no understanding of regulatory submissions and the documents produced for this process [29].

These results demonstrate that using regulatory data, such as the data from CSRs is rather new for the academic Cochrane community as a whole but researchers who are requesting or using regulatory documents to complete Cochrane Reviews consider access to these documents important and valuable for their analyses. Results of the survey also suggested that the availability of further guidance on how to interpret and use regulatory data in secondary analyses would help to promote the use of CSRs in Cochrane Reviews [29]. Conference proceedings have been presented regarding advantages and disadvantages in using CSRs versus published articles for Cochrane reviews, highlighting the extra information and opportunities of such data sources as well as challenges working with CSRs for Cochrane authors [30, 31]. Guidance of when to use Clinical Study Reports and other regulatory documents in systematic reviews has been developed as part of a ‘Cochrane Methods Innovation Fund’ [21].

### Potential impact of EMA policy 0070 on data utility of CSRs: academic author interviews

The examples considered within this review are based on CSRs and other regulatory documents (such as protocols, case report forms etc.) that were gathered before the implementation of EMA Policy 0070.

In order to further explore the use of CSRs in the projects and the potential impact that EMA Policy 0070 may have on the data utility of anonymised CSRs in academic research, in August to September 2017, we attempted to make contact with the authors of the journal publications identified and discussed within this review (Table 1 and Additional file 1: Table S1). Full details of the correspondence with authors who responded to us is provided in Additional file 2, shared with the permission of the authors.

In summary, all of the authors stated that their analyses would not have been possible without access to CSRs. None of the authors raised any specific concerns about anonymised or redacted CSRs (in line with EMA Policy 0070). In fact, one research team had used CSRs publicly available from a sponsor website which were redacted and this redaction did not impact upon the analysis from the author’s recollection [8]. Furthermore, none of the authors stated that their team had any difficulties in interpreting the information from the CSRs (a potential barrier raised within the Cochrane Survey) [20, 29]; the only problems related to ‘illegible’ text or the portable document format of the documents which prevented electronic searching.

All of the authors stated that some or all of their analyses or research would not have been possible if narratives and/or appendices (with participant listings) were removed from anonymised CSRs under EMA Policy 0070. One author stated that: “*Anonymised CSRs are ok, but the current EMA policy redacts important information about when the adverse events appeared as well as what they were. Newer CSRs do not have individual adverse event listings and the EMA are not even in possession of them.”*

Another author stated that: “*I have actually looked at data that are released under the EMAs new EMA Policy 0070, and they do provide fully redacted CSRs. So yes, I would say you could use these provided the drug is centrally licenced. But redactions may permit what data can be used, and they may not be of use for creating IPD datasets without the subject IDs and other patient-level information.”*

It should be emphasised that these observations are anecdotal and rhetorical as these projects were based on CSRs that were obtained before the implementation of EMA Policy 0070. We must also note that the quotes above reflect the opinions of the authors and we have not verified the accuracy of any statements made by the authors. However, our observations as well as the observations and views of the academic authors, and the rationales for the type of analyses being conducted using CSRs do raise some potential issues relating to ‘data utility’ of documents anonymised under EMA Policy 0070 (Phase 1). The full extent and any impact of such issues will not become apparent until sufficient research projects are conducted and published using anonymised CSRs prepared in line with EMA Policy 0070.

## Discussion

### Summary of findings and implications

CSRs have previously been considered as an ‘untapped’ source of detailed information relating to design, conduct and analysis of clinical trials [1]. The value of the information within CSRs is becoming increasingly recognised within the academic research community, particularly within the Cochrane Collaboration [20, 21, 29].

Publicly available summary data of clinical trial results from journal publications and trial registries may be suitable and sufficient to support some types of secondary analyses. However, an anonymised CSR provides complete information and data on study design and statistical methods, interpretation of results and the full set of endpoints’ results and statistics. Such a resource would certainly allow a more in depth appraisal including verification of numerical results, detailed assessment relating to bias such as selective outcome reporting and the conduct of novel analyses, such as systematic reviews and meta-analyses using data from all endpoints.

EMA Policy 0070 ‘Phase 1’, where anonymised CSRs are made public, is likely to further facilitate the secondary use of the information within CSRs for appraisal and secondary analysis. However, little consideration has been given to the data utility of the anonymised information within CSRs under EMA Policy 0070. The objective of this review was to investigate the type of research purposes and research methodologies employed in previous work using data from CSRs in academic research, and to hypothesize the impact of EMA Policy 0070 on the ‘data utility’ of future research of this kind.

Based on the number of requests made under EMA Policy 0043, we anticipate that academic researchers or research groups and the Pharmaceutical Industry are likely to be the primary recipients of anonymised CSRs under EMA Policy 0070. The research examples we discuss within this review indicate that the objectives and scopes of secondary analyses and novel research that have been conducted using CSR data are vast (Table 1, Additional file 1: Table S1). Authors of such research have communicated with us their concerns over the type of research that could be conducted in the future if information such as participant listings or narratives are redacted or removed completely under EMA Policy 0070 (Additional file 2).

Keeping the same conclusions and comparable numerical results of all primary, secondary and safety endpoints in the Anonymised CSRs to those of the original CSR (prior to any anonymisation) is of utmost importance. There are examples in the literature on how narratives are used to verify safety conclusions (see Additional file 1: Table S1). Handling of narratives, together with the handling of in-text listings, seems to be the most challenging aspect of EMA Policy 0070 from a technical standpoint and various levels of or approaches to anonymisation would further define different levels of data utility.

Certain free-text fields such as e.g. Adverse Events Reported Terms has been shown to be instrumental for certain secondary analysis to e.g. verify dictionary coding and conduct re-analysis [12–14]. Further, preserving Subject IDs and Dates in an anonymised format in order to follow the events of a participant, using sequences and distances to further understand the drug safety profile. An ongoing review of CSRs published under EMA Policy 0070 indicates that free-text variables (such as narratives) tend to be fully redacted when a dictionary-coded variable is available and deemed to be better suited for analysis [25]. The PhUSE De-Identification standard [32] recommends as a primary rule in the case of pro-active release of data to follow such rational and a secondary rule to “Review and redact PII” in such free-text variables. It is therefore advised to researchers to make it clear in their requests in the context of their research objective whether certain free-text variables (with all PII redacted) are required, even if a dictionary-coded variable is available in the given data domain.

Two main general types of analysis emerged from this research: Appraisal and Secondary Analysis. The different objectives across these two general analysis types should help prioritise anonymisation methods from a data utility perspective in addition to data privacy considerations. The classification of research objectives also provides more guidance for developing a qualitative approach to assess and document data utility of anonymised CSRs in-line with EMA Policy 0070 [17], Health Canada Public Release of Clinical Information Policy [33] or other contexts.

Further understanding of the safety profile of the drugs and verification of how conclusions of clinical studies are derived would certainly provide added value for many stakeholders and data consumers, including patients themselves. However, several journal publications that were reviewed within this review and described in Additional file 1: Table S1 have had their findings challenged by the sponsoring pharmaceutical companies through comments on journal websites. Discussion of academic findings and interpretations should always be encouraged but there is a risk that ‘rapid-response’ additional analyses as a challenge to published research may confuse readers and secondary data consumers such as clinical practitioners, patients and patient associations who cannot interpret which of the many published results are the ‘correct’ ones. Bonini et al. 2014 [34] also note that *“access to clinical data imposes a high ethical standard on anyone using those data, lest inappropriate reanalyses breed unjustified concern about the efficacy or safety of marketed drugs.”* We (SJN and JMF) are of the opinion that communication (and potentially collaboration) between academic research groups and pharmaceutical companies regarding interpretations of regulatory documents such as protocols, statistical analysis plans and CSRs, and the interpretation of results of secondary analyses from their different perspectives during the research projects should be encouraged. Such communication and discussions occurring before any results of secondary analyses are published within journals are likely to provide the most informative novel results and in turn, provide the most benefit to readers and data consumers.

### Limitations and future considerations

It must be emphasised that the examples of academic research using CSRs summarised within this review (Table 1 and Additional file 1: Table S1) are a selective sample and do not necessarily represent all research objectives which would make use of anonymised CSRs under EMA Policy 0070. Further, most observations provided to us by the authors of this research and our interpretations are rhetorical and speculative, rather than based on direct experience of anonymised CSRs published under EMA Policy 0070 and the validity of these observations may not become clear for some time.

The planned ‘Phase 2’ of EMA Policy 0070 which extends to sharing of individual participant data (IPD) should provide the next level of data utility that is required to conduct robust secondary analyses. A number of sponsors already provide access to anonymised IPD via data sharing platforms [22, 26] based on research requests for studies under the European Federation of Pharmaceutical Industries and Associations (EFPIA) principle of responsible clinical trial data sharing [35]. ‘Phase 2’ of EMA Policy 0070, when in effect, should in principle standardise the access to anonymised IPD for studies part of a central application in European Union regardless of the outcome of the application. The current needs of the research community which may include access to individual participant information such as full patient listings, which is out of scope of EMA Policy 0070 ‘Phase 1’, will be better addressed in ‘Phase 2’ of the policy where Individual Patient Datasets are in scope.

In the context of EMA Policy 0070, where anonymised CSRs are made public, a myriad of data recipient groups could be considered together with various objectives for reviewing and using the information within these anonymised CSRs. Their needs could differ from the academic research community and could be worth investigating at a later point.

In addition, at the time of writing, other regulatory agencies are defining, finalising and publishing their data transparency initiatives. FDA made an announcement in January 2018 [36] and started pilots with pharmaceutical companies where redaction is the only anonymization method and full listings of participant narratives are out-of-scope [37]. Health Canada started developing regulations around public access to clinical documents in 2017 and released a draft guidance for review in the second quarter of 2018 [33]. Difference of requirements between EMA Policy 0070 guidance, FDA and Health Canada approaches (under development) [38] may also lead to different anonymized versions of the same document in the public domain. Only when all policies are finalised will it become clear which versions under which jurisdiction serve best the needs of the academic community and others.

## Conclusions

In conclusion, EMA Policy 0070 guidance [17] refers to various levels of anonymisation but based only on level of risk of re-identification of participants without reference to different levels of data utility. The extent of the data utility of CSRs published under EMA Policy 0070 for academic research may not be fully known for some time, therefore in this interim time period, this review provides an initial insight into the previous use of CSR data, current practices for including regulatory data in academic research such as Cochrane systematic reviews and some early indications for the potential use of the data, and therefore the utility of anonymised data, from CSRs published under EMA Policy 0070.

## Supplementary information

**Additional file 1: Table S1.** Examples of academic work using CSRs in secondary research.

**Additional file 2.** Correspondence with authors of journal publications using CSRs for academic research. A list of semi-structured interview questions posed to corresponding authors of journal publications using CSRs for academic research and corresponding author responses to questions.

## Data Availability

All data contributing to this manuscript was extracted from articles within the public domain (see reference list) or taken from semi-structured interviews (see Additional file 1 for interviews). No additional unpublished data was collected.

## Abbreviations

AE: Adverse Event
CCI: Company Confidential Information
ClinicalTrials.gov: US Clinical Trials Database
CSR: Clinical Study Report
DIA: Drug Information Association
EFPIA: European Federation of Pharmaceutical Industries and Associations
EMA Policy 0043: European Medicines Agency Policy on access to documents (related to medicinal products for human and veterinary use)
EMA Policy 0070: European Medicines Agency policy on the publication of clinical data for medicinal products for human use (Phase I for Clinical Study Reports)
EMA: European Medical Agency
EU: European Union
EudraCT: European Clinical Trials Database
FDA: Food and Drug Administration
HTA: Health Technology Assessment
IPD: Individual Patient Data
LPLV: Last Patient Last Visit
OECD: Organization for Economic Co-operation and Development
PII: Personal Identifying Information
SAE: Serious Adverse Event
SAP: Clinical Trial Statistical Analysis Plan
