# Supplementary material for: European Medicines Agency Policy 0070: an exploratory review of data utility in clinical study reports for academic research
Source: BMC Med Res Methodol. 2019 Nov 5;19:204. doi: 10.1186/s12874-019-0836-3 (PMC6833240; doi:10.1186/s12874-019-0836-3)
Supplement: Supplementary file 1 — Additional file 1: Table S1. Examples of academic work using CSRs in secondary research. [file 12874_2019_836_MOESM1_ESM.doc]

**Table S1** Examples of academic work using CSRs in secondary research

| **Drug under consideration and references** | **Regulatory data Source(s)** | **Methods** | **Journal Publication Conclusion^1^** |
| --- | --- | --- | --- |
| **Reboxetine**  Eyding *et al* [1]  (see also additional references [2-4] from the same research group) | 13 CSRs and other regulatory documents from published and unpublished trials provided by Pfizer | - Appraisal: data and method comparison   *(CSRs and protocols compared to trial publications, trial registries and regulatory authority websites).*   - Data extraction - Systematic review and meta-analysis (novel) - AE counts | - Published data overestimated the benefit of reboxetine versus placebo by up to 115% and reboxetine versus SSRIs by up to 23%, and also underestimated harm. - Reboxetine is, overall, an ineffective and potentially harmful antidepressant. Published evidence is affected by publication bias, underlining the urgent need for mandatory publication of trial data. |
| **Paroxetine and imipramine**  Le Noury *et al* [5] | Individual participant data, CSR and Appendices available on GSK website and additional appendices provided by GSK for one trial (Study 329) | - Appraisal: Data and method comparison   *(IPD, CSR and original analysis compared).*   - Re-analysis (novel) - AE counts | - Neither paroxetine nor high dose imipramine showed efficacy for major depression in adolescents, and there was an increase in harms with both drugs. - Access to primary data from trials has important implications for both clinical practice and research, including that published conclusions about efficacy and safety should not be read as authoritative. - The reanalysis of Study 329 illustrates the necessity of making primary trial data and protocols available to increase the rigour of the evidence base. |
| **Orlistat**  Schroll *et al* [6] | 7 CSRs provided by Roche | - Appraisal: Data and method comparison   *(CSRs compared to protocols and trial publications).*   - AE counts | - For one trial, an additional 1,318 adverse events were identified that were not listed or mentioned in the CSR itself but could be identified through manually counting individual adverse events reported in an appendix. The majority of patients had multiple episodes of the same adverse event that were only counted once, though this was not described in the CSRs. - In the orlistat trials, we identified important disparities in the reporting of adverse events between protocols, clinical study reports, and published papers. Reports of these trials seemed to have systematically understated adverse events. Based on these findings, systematic reviews of drugs might be improved by including protocols and CSRs in addition to published articles. |
| **Orlistat**  Hodkinson *et al* [7] | 5 CSRs provided by Roche | - Appraisal: Data and method comparison   *(CSRs compared to trial publications).*   - AE counts | - Journal publications provided insufficient information on harms outcomes of the Orlistat trials and did not specify that a subset of harms data were being presented. - CSRs often present more complete data on harms, including serious adverse events - CSRs could support a more complete, accurate, and reliable investigation, and researchers undertaking evidence synthesis of harm outcomes should not rely only on incomplete published data that are presented in the journal publications. |
| **Oseltamvir**  Jefferson *et al* [8] | 83 CSRs obtained from EMA and Roche, of which 23 were used in the systematic review and meta-analysis | - Appraisal: Data and method comparison - *publication*Data extraction - Systematic review and meta-analysis (novel) - AE counts | - This is a report of the first Cochrane review to be based on all relevant full clinical study reports of a drug, augmented by regulatory comments. - The trade-off between benefits and harms should be borne in mind when making decisions to use oseltamivir for treatment, prophylaxis, or stockpiling. |
| **Duloxetine**  Maund *et al* [9-11] | 9 CSRs and protocols as appendices obtained from the EMA. | - Appraisal: Data and method comparison   *(CSRs compared to protocols, trial publications and clinicaltrials.gov entries).*   - Data extraction - Systematic review and meta-analysis (novel) - AE counts | - CSRs contained extensive data on harms that were unavailable in journal articles and trial registry reports. There were inconsistencies between protocol and CSRs. - The listings of adverse events for individual patients and narratives of adverse events within CSRs can provide additional information, including original investigator reported adverse event terms, which can enable a more accurate estimate of harms. - Following re-analysis using data from CSRs, the apparent harms of Duloxetine outweigh the benefits |
| **Gabapentin**  Vedula *et al [12]* | 20 CSRs (and other regulatory documents such as protocols) provided by Pfizer and Parke-Davis | - Appraisal: Data and method comparison   *(CSRs and other regulatory documents compared to published trial publications).* | - For 8 of the 12 trials reported as trial publications, the primary outcome differed in the published trial compared to the CSRs / protocol - Other sources of disagreement between published trials and regulatory documents included introduction of a new primary outcome, failure to distinguish between primary and secondary outcomes, relegation of primary outcomes to secondary outcomes and failure to report one or more protocol-defined primary outcomes. - This selective reporting of off-label use of gabapentin which threatens the validity of evidence for the effectiveness of off-label interventions |
| **Mannito**l  Nevitt *et al [13]* | CSR appendices of four trials and protocols, in addition to assistance with interpretation of CSR data, provided by manufacturer Pharmaxis. | - Appraisal: Data and method comparison   *(CSR data compared to published trial publications - assessment of reporting bias required for the Cochrane review)*   - Data extraction - Systematic review and meta-analysis (novel) - AE counts | - Detailed consideration of Quality of Life (an outcome important to patients with Cystic Fibrosis) would not have been possible without the additional CSR data provided - No consistent differences (low quality evidence) in any domain of quality of life found in any trial or any meta-analysis - Indication of an initial reduction in ‘burden of treatment’ of Mannitol compared to control at 4 months which was not maintained at 6 months. - Significant improvements in lung function up to and including 2 months, 4 months and 6 months (additional results provided from longitudinal model). |

1. *Some of the findings of these research papers have been challenged by the manufacturing companies of the interventions in question and those performing original research. In these cases, responses to publications have been published on the journal website.*

**References**

1. Eyding D, Lelgemann M, Grouven U, Harter M, Kromp M, Kaiser T, Kerekes MF, Gerken M, Wieseler B: **Reboxetine for acute treatment of major depression: systematic review and meta-analysis of published and unpublished placebo and selective serotonin reuptake inhibitor controlled trials.** *Bmj* 2010, **341:**c4737.

2. Wieseler B, Kerekes MF, Vervoelgyi V, McGauran N, Kaiser T: **Impact of document type on reporting quality of clinical drug trials: a comparison of registry reports, clinical study reports, and journal publications.** *Bmj* 2012, **344:**d8141.

3. Wieseler B, Wolfram N, McGauran N, Kerekes MF, Vervolgyi V, Kohlepp P, Kamphuis M, Grouven U: **Completeness of reporting of patient-relevant clinical trial outcomes: comparison of unpublished clinical study reports with publicly available data.** *PLoS Med* 2013, **10:**e1001526.

4. Kohler M, Haag S, Biester K, Brockhaus AC, McGauran N, Grouven U, Kolsch H, Seay U, Horn H, Moritz G, et al: **Information on new drugs at market entry: retrospective analysis of health technology assessment reports versus regulatory reports, journal publications, and registry reports.** *Bmj* 2015, **350:**h796.

5. Le Noury J, Nardo JM, Healy D, Jureidini J, Raven M, Tufanaru C, Abi-Jaoude E: **Restoring Study 329: efficacy and harms of paroxetine and imipramine in treatment of major depression in adolescence.** *Bmj* 2015, **351:**h4320.

6. Schroll JB, Bero L, Gotzsche PC: **Searching for unpublished data for Cochrane reviews: cross sectional study.** *Bmj* 2013, **346:**f2231.

7. Hodkinson A, Gamble C, Smith CT: **Reporting of harms outcomes: a comparison of journal publications with unpublished clinical study reports of orlistat trials.** *Trials* 2016, **17:**207.

8. Jefferson T, Jones MA, Doshi P, Del Mar CB, Hama R, Thompson MJ, Spencer EA, Onakpoya I, Mahtani KR, Nunan D, et al: **Neuraminidase inhibitors for preventing and treating influenza in healthy adults and children.** *Cochrane Database Syst Rev* 2014**:**Cd008965.

9. Maund E, Tendal B, Hrobjartsson A, Jorgensen KJ, Lundh A, Schroll J, Gotzsche PC: **Benefits and harms in clinical trials of duloxetine for treatment of major depressive disorder: comparison of clinical study reports, trial registries, and publications.** *Bmj* 2014, **348:**g3510.

10. Maund E, Tendal B, Hrobjartsson A, Lundh A, Gotzsche PC: **Coding of adverse events of suicidality in clinical study reports of duloxetine for the treatment of major depressive disorder: descriptive study.** *Bmj* 2014, **348:**g3555.

11. Maund E, Guski LS, Gotzsche PC: **Considering benefits and harms of duloxetine for treatment of stress urinary incontinence: a meta-analysis of clinical study reports.** *Cmaj* 2017, **189:**E194-e203.

12. Vedula SS, Bero L, Scherer RW, Dickersin K: **Outcome reporting in industry-sponsored trials of gabapentin for off-label use.** *N Engl J Med* 2009, **361:**1963-1971.

13. Nevitt SJ, Thornton J, Murray CS, Dwyer T: **Inhaled mannitol for cystic fibrosis.** *Cochrane Database Syst Rev* 2018, **2:**Cd008649.
