# Supplementary material for: European Medicines Agency Policy 0070: an exploratory review of data utility in clinical study reports for academic research
Source: BMC Med Res Methodol. 2019 Nov 5;19:204. doi: 10.1186/s12874-019-0836-3 (PMC6833240; doi:10.1186/s12874-019-0836-3)
Supplement: Supplementary file 2 — Additional file 2. Correspondence with authors of journal publications using CSRs for academic research. A list of semi-structured interview questions posed to corresponding authors of journal publications using CSRs for academic research and corresponding author responses to questions. [file 12874_2019_836_MOESM2_ESM.docx]

**Additional file 2: Appendix 1: Correspondence with authors of journal publications using CSRs for academic research**

During August 2017, an email was sent to the corresponding authors of all manuscripts using CSRs for academic research identified with the following questions:

1. Could you explain the rationale for using CSRs for your research (rather than published trial reports, IPD etc.)?
2. Were any specific outcomes reported within the CSRs for interest to you?
3. Which sections of the CSRs did you use for your research (e.g. summary tables, narratives, appendices, participant listings, others sections etc.)
4. If you used the narratives, can you explain exactly what you used them for? For example, did you wish to extract participant level information to create a dataset
5. Did you have any difficulties in extracting or interpreting information from the CSRs?
6. Were there any key findings of your research specifically relating to the use of CSRs?
7. Would you have been able to complete this research without access to CSRs?
8. Would you have been able to complete this research if narratives and/or appendices (with participant listings) had been removed from the CSRs?
9. Would you have been able to complete this research if you had been provided with anonymised CSRs (in line with EMA policy 0070)

Authors were invited to share their responses to the questions by email or to discuss their manuscripts and the use of CSRs in academic research with Sarah Nevitt (SN) on the telephone. Authors were also invited to share any additional comments outside of the questions above.

All authors who responded were aware that their responses would be used within the present manuscript and provided verbal or written consent to SN (further details available on request).

**Telephone conversation with Dr Jon Jureidini, 18 August 2017.**

Dr Jureidini was involved in the project relating to the manuscript:

*Le Noury J, Nardo JM, Healy D, et al. Restoring Study 329: efficacy and harms of paroxetine and imipramine in treatment of major depression in adolescence. BMJ 2015;351:h4320. doi: 10.1136/bmj.h4320*

Dr Jureidini spoke to SN regarding this project on 18 August 2017. This appendix summarises the key findings of this work with comments from Dr Jureidini (conversations summarised by SN, comments are not direct quotes from Dr Jureidini).

This was the first study published using data from CSDR. The authors also used CSRs which were available on the GSK website (including appendices) that had been redacted to some extent. The initial reporting of Study 329 has been considered controversial, hence this research team wished to perform a re-analysis with the original data and regulatory documents of Study 329. The original analysis found the drugs to show efficacy with limited harms (only significant adverse events reported). The re-analysis shows that neither drug actually showed any efficacy and there was an increase in harms from both drugs.

*Comment from Dr Jureidini (on the rationale for the work): Everyone is interested in efficacy of the drugs and p values. This is what the main papers always concentrate on and the harms are always a secondary consideration. We should be more interested in the harms of the data, these are never reported enough and it can be hard to get people interested in harms. To get a full picture of potential harms, you need to look a lot deeper into the data and the CSRs – this will never be fully captured in a trial publication.*

*There were clear issues with the initial publication of Study 329 – not fraudulent, the authors were very honest, almost too honest as it revealed that the efficacy they described was at odds with the data. This became even more evident looking at the CSRs.*

The use of CSRs in this project was for the harms data. The authors recoded some of the narrative descriptions of adverse events. Due to unclear descriptions within the publically available appendices from the GSK website, the authors requested an extra unpublished extra appendix of the study CRFs. This was provided via the CSDR remote data access system.

*Comment from Jureidini: The system was so cumbersome and difficult to access that due to the time involved of recoding and comparing adverse events, we only completed this task for around a third of AEs; the AEs we considered to be the most important. We were criticised for this by reviewers of the BMJ article but as we had no funding and limited resources for this analysis but didn’t have a lot of choice.*

*The redaction of information from narratives and appendices didn’t really cause any issues as far as I can recall. The problem was that some of the text was illegible – maybe due to the age of the document which wasn’t helped by the remote data access system which prevented zooming in, using software to improve quality etc.*

*We had no problem with the content or interpreting the documents etc. The problems were with the quality of the documents and trying to use them in the remote system.*

*This analysis would have been impossible without access to the appendices and it would be ‘travesty’ if the implication of EMA Policy 0070 means that appendices are completely redacted or no longer shared.*

**Correspondence with Dr Beate Wieseler, 20 August 2017**

Questions were sent to Dr Wieseler by SN to further explore the use of CSRs in the project relating to the manuscript:

*Eyding D, Lelgemann M, Grouven U, et al. Reboxetine for acute treatment of major depression: systematic review and meta-analysis of published and unpublished placebo and selective serotonin reuptake inhibitor controlled trials. BMJ 2010;341:c4737. doi: 10.1136/bmj.c4737*

Dr Wieseler provided the following references which also report the experiences of the research group using CSRs (2-4):

*Wieseler B, Wolfram N, McGauran N, et al. Completeness of Reporting of Patient-Relevant Clinical Trial Outcomes: Comparison of Unpublished Clinical Study Reports with Publicly Available Data. PLOS Medicine 2013;10(10):e1001526. doi: 10.1371/journal.pmed.1001526*

*Wieseler B, Kerekes MF, Vervoelgyi V, McGauran N, Kaiser T. Impact of document type on reporting quality of clinical drug trials: a comparison of registry reports, clinical study reports, and journal publications. BMJ. 2012 Jan 3;344:d8141.*

*Köhler M, Haag S, Biester K, Brockhaus AC, McGauran N, Grouven U, Kölsch H, Seay U, Hörn H, Moritz G, Staeck K. Information on new drugs at market entry: retrospective analysis of health technology assessment reports versus regulatory reports, journal publications, and registry reports. BMJ. 2015 Feb 26;350:h796.*

Dr Wieseler also provided the following responses to the questions by e-mail and is happy for the responses to be reported in this paper:

10. Could you explain the rationale for using CSRs for your research (rather than published trial reports, IPD etc.)?

We are interested in the full evidence base of the interventions under assessment in our Health Technology Assessment reports to be able to provide unbiased assessments for decision making in the German health care system. By using regulatory documents like CTD clinical overviews and clinical summaries and specifically CSRs we want to overcome publication and outcome reporting bias.

Specifically we need full information on trial methods including the study protocol with any amendments and the statistical analysis plan (SAP) to be able to understand the study planning or any changes in the conduct of the study or the analyses. Among other things we use this information to choose relevant endpoints/analyses and assess the risk of bias on a study or endpoint level. Journal publications and registry reports do not provide the level of detail we need. E.g. journal publications do not provide full inclusion/exclusion criteria or full specification of endpoints or statistical analyses (including information on handling of missing or specification of statistical methodology like models used in the analyses.)

Furthermore, we need full numerical summary data on all endpoints (including information on which endpoints/analyses were pre-specified or defined post-hoc) to be able choose the endpoints/analyses relevant to our assessment and to conduct meta-analyses. Often numerical information in journal publications is too limited for meta-analysis. We are also interested in subgroup or sensitivity analyses which also might not be presented in documents other than the CSR.

11. Were any specific outcomes reported within the CSRs for interest to you?

We are specifically interested in patient-relevant outcomes (i.e. outcomes describing how a patient feels, functions or survives). Often these endpoints are secondary endpoints which are not fully published in journals. Our research has described and quantified to which extent we could add endpoints from CSRs as compared to other sources (BMJ 2012;344:d8141, PLoS Med 10(10): e1001526; BMJ 2015;350:h796)

12. Which sections of the CSRs did you use for your research (e.g. summary tables, narratives, appendices, participant listings, others sections etc.)

We use the full CSR including end-of-text tables and appendices for our work.

13. If you used the narratives, can you explain exactly what you used them for? For example, did you wish to extract participant level information to create a dataset

We rarely use narratives because the information we use in most cases is available also in other data presentations (e.g. listings or summary tables of SAEs). However, if these other data presentations are missing, we might extract information from narratives.

14. Did you have any difficulties in extracting or interpreting information from the CSRs?

We do not have difficulties in extracting or interpreting information from well written CSRs.

15. Were there any key findings of your research specifically relating to the use of CSRs?

The CSRs of reboxetine allowed to assess all evidence available in a situation where only a biased set of studies was published in journals (please see BMJ 2010;341:c4737). We have been able to extract substantially more methodological and endpoint information for our assessments from CSRs than from other sources (please see references given under Question 2). Futhermore, often we were only able to understand a study included in our assessment fully by using the CSR including the protocol and SAP (please see Z Evid Fortbild Qual Gesundhwes. 2017 Feb;120:3-8 for a description of the example of our assessment of liraglutide).

16. Would you have been able to complete this research without access to CSRs?

Without CSRs we would have missed important information.

17. Would you have been able to complete this research if narratives and/or appendices (with participant listings) had been removed from the CSRs?

We definitely do need appendices including protocols and amendments and the SAP. If we need participant listings depends on the open questions that arise from the assessment (see e.g. the assessment of liraglutide in which we needed the information of when hypoglycemic events occurred in individual patients).

18. Would you have been able to complete this research if you had been provided with anonymised CSRs (in line with EMA policy 0070)?

In rare cases I can forsee that we need to follow study participants through several listings. This would only be possible if there is some kind of ID (not necessarily the original ID from the study).

**Correspondence with Dr Jeppe Schroll, 31 August 2017**

Questions were sent to Dr Schroll by SN to further explore the use of CSRs in the project relating to the manuscript:

*Schroll JB, Bero L, Gotzsche PC. Searching for unpublished data for Cochrane reviews: cross sectional study. BMJ 2013;346:f2231. doi: 10.1136/bmj.f2231*

Dr Schroll provided the following responses by e-mail and is happy for the responses to be reported in this paper:

1. Could you explain the rationale for using CSRs for your research (rather than published trial reports, IPD etc.)?

The purpose of our paper was to compare published with unpublished data.

2. Were any specific outcomes reported within the CSRs for interest to you?

We covered reported adverse event and methods on how to handle adverse events. Lots of outcomes were reported that were not available in the publications

3. Which sections of the CSRs did you use for your research (e.g. summary tables, narratives, appendices, participant listings, others sections etc.)

We used summary tables, appendices and narratives and participant listings. For our CSR individual patient listings of adverse events were available.

4. If you used the narratives, can you explain exactly what you used them for? For example, did you wish to extract participant level information to create a dataset

We explored the reason for discontinuation and found divergence from the listing of withdrawals. In the narratives the sponsors’ causality assessment was available.

5. Did you have any difficulties in extracting or interpreting information from the CSRs?

It was a great difficult that the documents were not in a “text readable” format. Which made electronic searching impossible. The pages had more than one page number and there was – in out material – no overall table of contents. Only for the specific sections.

6. Were there any key findings of your research specifically relating to the use of CSRs?

We found that a lot of data is left out of the publications and that important limitations and assumptions were not described.

7. Would you have been able to complete this research without access to CSRs?

No

8. Would you have been able to complete this research if narratives and/or appendices (with participant listings) had been removed from the CSRs?

No

9. Would you have been able to complete this research if you had been provided with anonymised CSRs (in line with EMA policy 0070)

Anonymised CSRs are ok, but the current EMA policy redacts important information about when the adverse events appeared as well as what they were. Newer CSRs does not have individual adverse event listings and the EMA are not even in possession of them.

**Correspondence with Professor Catrin Tudur Smith and Dr Alex Hodkinson, September 2017**

Questions were sent to Professor Tudur Smith (CTS) and Dr Hodkinson (AH) by SN to further explore the use of CSRs in the project relating to the manuscript:

*Hodkinson A, Gamble C, Smith CT. Reporting of harms outcomes: a comparison of journal publications with unpublished clinical study reports of orlistat trials. Trials 2016;17(1):1-11. doi: 10.1186/s13063-016-1327-z*

Professor Tudur Smith (CTS) and Dr Hodkinson (AH) provided the following responses by e-mail and is happy for the responses to be reported in this paper:

19. Could you explain the rationale for using CSRs for your research (rather than published trial reports, IPD etc.)?

CTS: As a methodological exercise to compare the information available in CSR s versus information available in published journal articles

AH: Really this was just a methodological piece of work displaying the value of CSRs when there evidence for underreporting of harms in published literature.

20. Were any specific outcomes reported within the CSRs for interest to you?

CTS: Adverse events only

AH: All harm outcomes (AEs and SAEs) but also the timing of events, grading and safety narratives if reported

21. Which sections of the CSRs did you use for your research (e.g. summary tables, narratives, appendices, participant listings, others sections etc.)

CTS: We didn’t receive narratives but this may have provided additional information. The main source of information we used were the IPD listings of adverse events and tables

AH: Roche I believe have changed the format of their CSRs since this piece of work, but data were primarily obtained from the following sections:

- Module I (core report which follows the ICH E3 format): sections 2.6 (safety parameters), 2.9.4 (Safety analysis), 3.4 (safety results).
- Modules II: glossary of original and MedDRA preferred terms for AEs
- Module V: section 5, Safety analysis plan

22. If you used the narratives, can you explain exactly what you used them for? For example, did you wish to extract participant level information to create a dataset

AH: No we didn’t use the safety narratives, as they were rarely reported. If they were reported consistently we may have been able to create an IPD dataset.

23. Did you have any difficulties in extracting or interpreting information from the CSRs?

CTS: Large documents that are difficult to navigate at first but they were well structured and so quite straightforward to locate the information

AH: Not really, other than Roche had removed a number of pages in one of the CSRs and redacted quite a lot of information throughout. We did contact them about this, apparently there was some confidential information on these pages. I wasn’t convinced.

24. Were there any key findings of your research specifically relating to the use of CSRs?

CTS: We found additional information and additional detail available in the CSRs but also additional time required to extract the information

AH: Obviously there were high numbers of AE and particularly SAEs that were not reported in the publication (see bar graphs in paper), and 5 statistically significant harm outcomes were detected using the CSRs.

25. Would you have been able to complete this research without access to CSRs?

CTS: No

AH: Due to the underreporting of harms data in publications probably not. CSRs are far more detailed than publications, and I would recommend they are used where possible for synthesis of harms.

26. Would you have been able to complete this research if narratives and/or appendices (with participant listings) had been removed from the CSRs?

AH: N/A as we didn’t use the safety narratives, and the participant listing of AEs in Module IV of the report were removed.

27. Would you have been able to complete this research if you had been provided with anonymised CSRs (in line with EMA policy 0070)

CTS: Possibly

AH: I have actually looked at data that are released under the EMAs new policy 70, and they do provide fully redacted CSRs. So yes, I would say you could use these provided the drug is centrally licenced. But redactions may permit what data can be used, and they may not be of use for creating IPD datasets without the subject IDs and other patient-level info.
